# Complete genome sequence of an endophytic bacterium *Enterobacter vonholyi* strain IBGEEm25P6 from wheat seeds

**DOI:** 10.1128/mra.00376-26

**Published:** 2026-06-30

**Authors:** Dipali Rani Gupta, Lamya Muazzeda Medha, Sharmin Islam, Avi Kumar Badhon, M. Nazmul Hoque, Tofazzal Islam

**Affiliations:** 1 Institute of Biotechnology and Genetic Engineering (IBGE), Gazipur Agricultural University (GAU) 198780, Gazipur, Bangladesh; 2 Molecular Biology and Bioinformatics Laboratory, Department of Gynecology, Obstetrics and Reproductive Health, GAU 198780 , Gazipur, Bangladesh; University of Strathclyde, Glasgow, United Kingdom

**Keywords:** seed endophyte, antifungal, nitrogen fixation, Oxford Nanopore sequencing, wheat blast resistance

## Abstract

We report the complete genome sequence of *Enterobacter vonholyi* strain IBGEEm25P6, an endophyte isolated from wheat seeds. The 4.7-Mb genome is predicted to encode diverse biosynthetic genes putatively associated with nutrient metabolism and antifungal activity, including genes predicted to be involved in nitrogen fixation.

## ANNOUNCEMENT

The members of the genus *Enterobacter* are widely distributed across diverse environments, including soil, water, plants, and animals. Certain *Enterobacter* species enhance plant growth by increasing nutrient and water bioavailability and by synthesizing growth-promoting hormones (1–4). Furthermore, they possess the potential to suppress phytopathogens through the production of volatile and non-volatile secondary metabolites (5). The isolate was obtained from seeds of blast-resistant wheat cultivar BARI Gom 33, collected in March–April 2024, from Gazipur Agricultural University experimental farm, Bangladesh (24.0362° N, 90.3959° E). Seeds were sterilized with 80% ethanol (10 min) and 1% NaOCl (5 min), followed by three sterile water rinses. A 1-g pooled sample was homogenized in 9 mL sterile water, serially diluted (10⁻⁵), plated on nutrient agar (Oxoid, UK), and was incubated at 27°C ± 2°C for 24 h. Distinct colonies were purified by repeated streaking based on morphology (6). A single colony, designated IBGEEm25P6, was cultured in nutrient broth (Oxoid) at 27°C, 180 rpm for 24 h for genomic DNA extraction using a GeneJET kit (Thermo Fisher Scientific, USA), and DNA concentration was measured with a Qubit 4.0 fluorometer. Sequencing libraries were constructed from intact high-molecular-weight DNA using the Oxford Nanopore Technologies (ONT) Ligation Sequencing Kit (SQK.NBD.114.24) and barcoded with the Native Barcoding Kit 96 V14, following the manufacturer’s protocol. Sequencing was performed on a MinION Mk1B device. No DNA shearing or size selection was performed (7). Basecalling was done using Dorado v1.2.0 (SUP model, Phred > 7). Raw read quality was evaluated using NanoPlot v1.46.1 (8). The final data set, consisting of 16,592 raw reads with a mean length of 12,013.6 bp and an N50 of 21,644, was trimmed and filtered (Q > 15, length >1,000 bp) using NanoFilt v2.8.0 (9). *De novo* genome assembly was performed using Flye v2.9.5-b1801 (10), followed by polishing with Medaka v2.0.1 (11). Taxonomic identity was confirmed by NCBI ANI analysis (11), assigning IBGEEm25P6 to *Enterobacter vonholyi* (ANI, 98.14%; coverage, 90.96%; GCA_008364555.1). Genome annotation was carried out using the NCBI GenBank Prokaryotic Genome Annotation Pipeline (PGAP) v6.10 (12). Genome quality, including completeness and contamination, was assessed using CheckM v1.2.4 with *lineage_wf*, *Enterobacter* CheckM marker set (13). Default parameters were used for all software unless otherwise specified.

The gapless, unrotated (retained) assembly comprised two circular contigs totaling 4,656,279 bp (55.5% GC), including a complete circular chromosome of 4,542,623 bp and a 113,656-bp plasmid, consistent with Flye output. PGAP annotations revealed 4,344 CDSs, including 4,308 protein-coding genes, 83 tRNAs, and 25 rRNAs (Table 1). Functional annotation predicted genes putatively associated with nitrogen fixation, phosphorus solubilization, potassium uptake/regulation, indicating potential roles in nutrient mobilization. The genome is also predicted to encode genes linked to siderophore production, iron transport, acetoin biosynthesis, motility, and chemotaxis, commonly associated with biocontrol. Together, these features suggest metabolic versatility and potential plant-beneficial traits (14–17). Overall, the IBGEEm25P6 strain provides a useful resource for understanding its functional attributes, supporting further investigation into its biological roles.

**TABLE 1:** Genomic features of *E. vonholyi* strain IBGEEm25P6 isolated from seeds of the wheat cultivar BARI gom 33 from GenBank annotations

Genome feature	IBGEEm25P6
GenBank accession	JBSTPG000000000
Assembly accession	GCA_054889185.1
BioSample accession	SAMN53502475
SRA	SRR37387038
Number of reads	16,592 (N50: 21,644)
Assembled genome size (bp)	4,656,279
GC %	55.5
Coverage	38×
Genome completeness (%)	99.61
CheckM contamination (%)	0.31
N50 value (bp)	4,542,623
N90 value (bp)	4,542,623
Number of contigs	2
Size of circular chromosome (bp)	4,542,623
Genes (total)	4,459
CDSs (total)	4,344
Genes (coding)	4,308
CDSs (with protein)	4,308
Genes (RNA)	115
rRNAs	9, 8, 8 (5S, 16S, 23S)
Complete rRNAs	9, 8, 8 (5S, 16S, 23S)
tRNAs	83
ncRNAs	7
Pseudo genes (total)	36
CDSs (without protein)	36
Pseudo genes (ambiguous residues)	0 of 36
Pseudo genes (frameshifted)	24 of 36
Pseudo genes (incomplete)	22 of 36
Pseudo genes (internal stop)	10 of 36
Pseudo genes (multiple problems)	18 of 36

## Data Availability

The whole-genome sequence of *Enterobacter vonholyi* strain IBGEEm25P6 has been deposited in GenBank, BioProject database (Mining Bangladesh Biogold), and Sequence Read Archive (SRA) under accession numbers JBSTPG000000000, BioProject PRJNA1055760, and SRA SRR37387038, respectively.

## References

[1] Kumar A, Maurya BR, Raghuwanshi R, Meena VS, Tofazzal Islam M. 2017. Co-inoculation with *Enterobacter* and rhizobacteria on yield and nutrient uptake by Wheat (*Triticum aestivum* L.) in the alluvial soil under indo-gangetic plain of India. J Plant Growth Regul 36:608–617. doi:10.1007/s00344-016-9663-5

[2] Synek L, Rawat A, L’Haridon F, Weisskopf L, Saad MM, Hirt H. 2021. Multiple strategies of plant colonization by beneficial endophytic *Enterobacter* sp. SA187. Environ Microbiol 23:6223–6240. doi:10.1111/1462-2920.15747 34472197

[3] Islam MT, Deora A, Hashidoko Y, Rahman A, Ito T, Tahara S. 2007. Isolation and Identification of potential phosphate solubilizing bacteria from the rhizoplane of *Oryza sativa* L. cv. BR29 of Bangladesh. Zeitschrift für Naturforschung C 62:103–110. doi:10.1515/znc-2007-1-218 17425114

[4] Zouagui H, Zouagui R, Ibrahimi A, Sbabou L. 2025. Comparative genomics uncovers adaptive and biotechnological potential of *Enterobacter xiangfangensis* MDMC82 isolated from desert, and highlights putative core *Enterobacteriaceae* functions. Sci Rep 15:41234. doi:10.1038/s41598-025-25044-x 41271842 PMC12638922

[5] Gong A-D, Dong F-Y, Hu M-J, Kong X-W, Wei F-F, Gong S-J, Zhang Y-M, Zhang J-B, Wu A-B, Liao Y-C. 2019. Antifungal activity of volatile emitted from *Enterobacter asburiae* Vt-7 against *Aspergillus flavus* and aflatoxins in peanuts during storage. Food Control 106:106718. doi:10.1016/j.foodcont.2019.106718

[6] Dutta S, Khatun A, Gupta DR, Surovy MZ, Rahman MM, Mahmud NU, Emes RD, Warry A, West HM, Clarke ML, Hoque MN, Hossain MM, Salam MA, Islam MT. 2020. Whole-genome sequence of a plant growth-promoting strain, *Serratia marcescens* BTL07, isolated from the rhizoplane of *Capsicum annuum* L. Microbiol Resour Announc 9:e01484-19. doi:10.1128/MRA.01484-19 32354984 PMC7193939

[7] Sereika M, Kirkegaard RH, Karst SM, Michaelsen TY, Sørensen EA, Wollenberg RD, Albertsen M. 2022. Oxford Nanopore R10.4 long-read sequencing enables the generation of near-finished bacterial genomes from pure cultures and metagenomes without short-read or reference polishing. Nat Methods 19:823–826. doi:10.1038/s41592-022-01539-7 35789207 PMC9262707

[8] De Coster W, Rademakers R. 2023. NanoPack2: population-scale evaluation of long-read sequencing data. Bioinformatics 39:btad311. doi:10.1093/bioinformatics/btad311 37171891 PMC10196664

[9] De Coster W, D’Hert S, Schultz DT, Cruts M, Van Broeckhoven C. 2018. NanoPack: visualizing and processing long-read sequencing data. Bioinformatics 34:2666–2669. doi:10.1093/bioinformatics/bty149 29547981 PMC6061794

[10] Kolmogorov M, Bickhart DM, Behsaz B, Gurevich A, Rayko M, Shin SB, Kuhn K, Yuan J, Polevikov E, Smith TPL, Pevzner PA. 2020. metaFlye: scalable long-read metagenome assembly using repeat graphs. Nat Methods 17:1103–1110. doi:10.1038/s41592-020-00971-x 33020656 PMC10699202

[11] Oxford Nanopore Technologies. 2018. Oxford nanopore technologies medaka. https://github.com/nanoporetech/medaka.

[12] Tatusova T, DiCuccio M, Badretdin A, Chetvernin V, Nawrocki EP, Zaslavsky L, Lomsadze A, Pruitt KD, Borodovsky M, Ostell J. 2016. NCBI prokaryotic genome annotation pipeline. Nucleic Acids Res 44:6614–6624. doi:10.1093/nar/gkw569 27342282 PMC5001611

[13] Parks DH, Imelfort M, Skennerton CT, Hugenholtz P, Tyson GW. 2015. CheckM: assessing the quality of microbial genomes recovered from isolates, single cells, and metagenomes. Genome Res 25:1043–1055. doi:10.1101/gr.186072.114 25977477 PMC4484387

[14] Bruto M, Prigent-Combaret C, Muller D, Moënne-Loccoz Y. 2014. Analysis of genes contributing to plant-beneficial functions in plant growth-promoting rhizobacteria and related proteobacteria. Sci Rep 4:6261. doi:10.1038/srep06261 25179219 PMC4151105

[15] Sachman-Ruíz B, Wong-Villarreal A, Aguilar-Marcelino L, Lozano-Aguirre LF, Espinosa-Zaragoza S, Reyes-Reyes AL, Sanzón-Gómez D, Mireles-Arriaga AI, Romero-Tirado R, Rocha-Martínez MK, Pérez-de la Rosa JD, Sánchez-Cruz R, Gómez-Gutiérrez JA. 2022. Nematicidal, acaricidal and plant growth-promoting activity of *Enterobacter* endophytic strains and identification of genes associated with these biological activities in the genomes. Plants 11:3136. doi:10.3390/plants11223136 36432865 PMC9695364

[16] Xu J, Fang J, Wu R, Wang Y, Zhang C, Wang X, Qiu T. 2026. Exploring the genomic features and plant growth-promoting properties of *Enterobacter vonholyi* Y16 isolated from maize rhizosphere. Microbiol Res 305:128437. doi:10.1016/j.micres.2026.128437 41512536

[17] Zhang L, Chen W, Jiang Q, Fei Z, Xiao M. 2020. Genome analysis of plant growth-promoting rhizobacterium *Pseudomonas chlororaphis* subsp. aurantiaca JD37 and insights from comparasion of genomics with three pseudomonas strains. Microbiol Res 237:126483. doi:10.1016/j.micres.2020.126483 32402945

